# Breast Cancer Survival and Incidence: 10 Years Cancer Registry Data in the Northwest, Iran

**DOI:** 10.1155/2020/1963814

**Published:** 2020-05-01

**Authors:** Roya Dolatkhah, Mohammad Hossein Somi, Mohammad Asghari Jafarabadi, Mehrnaz Hosseinalifam, Sepideh Sepahi, Mina Belalzadeh, Marzieh Nezamdoust, Saeed Dastgiri

**Affiliations:** ^1^Hematology and Oncology Research Center, Tabriz University of Medical Sciences, Tabriz, Iran; ^2^Liver and Gastrointestinal Diseases Research Center, Tabriz University of Medical Sciences, Tabriz, Iran; ^3^Road Traffic Injury Research Center, Tabriz University of Medical Sciences, Tabriz, Iran; ^4^Tabriz University of Medical Sciences, Tabriz, Iran; ^5^Cancer Registry Office, Liver and Gastrointestinal Diseases Research Center, Tabriz University of Medical Sciences, Tabriz, Iran; ^6^Tabriz Health Services Management Research Center, Tabriz University of Medical Sciences, Tabriz, Iran

## Abstract

**Methods:**

Data were obtained from East Azerbaijan cancer registry database for the 10-year period between 2007 and 2016. Survival analysis was performed to calculate the breast cancer-specific survival proportions and mortality rates. Joinpoint trend analysis was performed to estimate the incidence trend of the cancer.

**Results:**

A total number of 4989 patients were recorded with primary diagnosis of breast cancer. Of them, we collected follow-up data for 1335 (1309 female and 26 male). The 10-year crude mortality rate was 3.34 (per 100,000). The one-, two-, three-, five-, and ten-year breast cancer-specific survival proportions were 0.92 (95% CI 0.91-0.93), 0.88 (95% CI 0.86-0.90), 0.84 (95% CI 0.83-0.86), 0.77 (95% CI 0.74-0.80), and 0.65 (95% CI 0.60-0.70), respectively. Over the study period, the age-standardized incidence rates increased from 21.68 to 36.99 (per 100,000) with an annual percentage change of 5.5 percent. Older individuals and males patients had significantly worse survival, and patients with high-grade tumors had significantly higher risk of mortality.

**Conclusion:**

A relatively better survival for breast cancer in East Azerbaijan, Iran, was observed compared to the overall breast cancer-specific survival proportions and mortality rates in the country. However, it is still poor compared to the developed countries indicating that inappropriate treatment modalities might have played a role on this.

## 1. Introduction

Breast cancer is remarkably common across the globe. In 2018, it was responsible for an estimated 2.1 million cancers accounting for the fifth leading cause of cancer deaths worldwide [1, 2]. One in every 9 women in developed countries and one in every 20 in less developed areas may have the risk of breast cancer [2]. Age-standardized incidence rate (ASIR) is now annually increasing by 29 percent in the world. This secular trend has been attributed to the changes in the population age structure (16 percent), population growth (12 percent), and the etiologic causes of the cancer (1 percent) [2].

Breast cancer is accounting for 12.5 percent of all cancers in Iran. It is the sixth leading cause of death in the country [1, 3]. According to the Iranian National Cancer Registry (INCR), the annual ASIR for primary breast cancer is 27.4 (per 100,000) with a crude incidence of 22.6 (per 100,000) [3, 4]. Although the burden of breast cancer is still low in the country, there has been an increasing trend for the incidence and mortality rates in recent years [5, 6].

There are contradictory reports about the breast cancer-specific survival proportions and mortality rates in various regions in Iran. However, the overall survival proportions are much lower than reported from developed countries [7–11].

The aim of this study was to report the breast cancer-specific survival proportions and mortality rates, and trend analysis of breast cancer between 2007 and 2016 in a northwest region in Iran.

## 2. Methods

### 2.1. Study Setting

The study area, East Azerbaijan, is located in the northwest of Iran. The center of the area is the city of Tabriz, one of the three major cities in Iran. Due to the role of this city as the capital in the area, individuals from various ethnic groups and religions live there. The public health system comprises a network of health centers and hospitals providing health care and medical services for the regional population. Health and medical facilities are all working under the Tabriz University of Medical Sciences. This is one of the five top universities in the country providing medical and health services for more than six million in the region. Most people live (nearly 70 percent) in the cities [Statistics & Administration Office, East Azarbaijan Province, National Census Data, 2019].

The East Azerbaijan Population-Based Cancer Registry (EA-PBCR) register/provide regional cancer data from 33 pathology laboratories, 20 private and university hospitals, radiotherapy and hematology clinics, and 35 imaging centers. Data related to the death in the area were obtained from regional bureaus of statistics.

### 2.2. Study Subjects and Data

Data for patients diagnosed with breast cancer were obtained from the EA-PBCR database for the 10-year period between 2007 and 2016. Breast cancer records were defined based on the standard coding system of the International Classification of Diseases (ICD)-Oncology under the codes C50.0–9 [12]. The stage or grade of a tumor was defined according to the World Health Organization classification of breast tumors [13].

The data related to the age at diagnosis, sex, morphology (i.e., histology, behavior, and grade), topography (i.e., the primary site of origin), stage, and grade were collected from EA-PBCR. Follow-up and outcome data were obtained by contacting patients/relatives, and from our Hospital Information System (HIS).

### 2.3. Statistical Analysis

Joinpoint software was used to estimate the breast cancer incidence trend, Annual Percentage Change (APC) and Average Annual Percentage Change (AAPC) (the software is available from https://surveillance.cancer.gov/joinpoint/). The ASIR for breast cancer (per 100,000) was estimated using the standard world population for the year 2000. For the breast cancer-specific survival proportions and mortality rates, the survival status of patients was recorded from the date of diagnosis to the date of death due to the breast cancer. Patients who remained alive until the last follow-up were censored. The maximum follow-up time in this study was ten years. The survival analysis was performed using the Kaplan-Meier method. Log-rank test and Cox regression were computed to examine the survival status by age, sex, and morphology and grade using the STATA MP 14.2 (Stata Corp LP, College Station, Texas 77845 USA).

## 3. Results

The characteristics of 4989 patients with breast cancer are presented in Table 1. The study subjects included 4885 females (97.9 percent), 104 males (2.1 percent), with an average age of 50.4 (SD = 12.9) ranging between 21 and 98 years. Most patients were diagnosed with ductal carcinoma (62.1 percent) followed by lobular carcinoma (11.7 percent). In terms of tumor grade, 736 cases (14.8 percent) had grade I, 1348 cases (27.0 percent) grade II, and 248 cases (4.9 percent) grade III tumor.

We collected the follow-up data for 1335 over the ten years' follow-up in this study mostly including females (98.1 percent) with a mean age of 50.6 years. The majority of patients were in grade II tumor class (38.3 percent) (Table 1).

### 3.1. Breast Cancer-Specific Survival Analysis Results

The median follow-up time was 47.5 months. The breast cancer-specific ten-year crude mortality rate was 3.3 (per 100,000). The mean and median survivals were 57.4 and 47.5 months, respectively. The one-, two-, three-, five-, and ten-year breast cancer-specific survival proportions were 0.92 (95% CI 0.91-0.93), 0.88 (95% CI 0.86-0.90), 0.84 (95% CI 0.83-0.86), 0.77 (95% CI 0.74-0.80), and 0.65 (95% CI 0.60-0.70), respectively.

**Figure 1:**
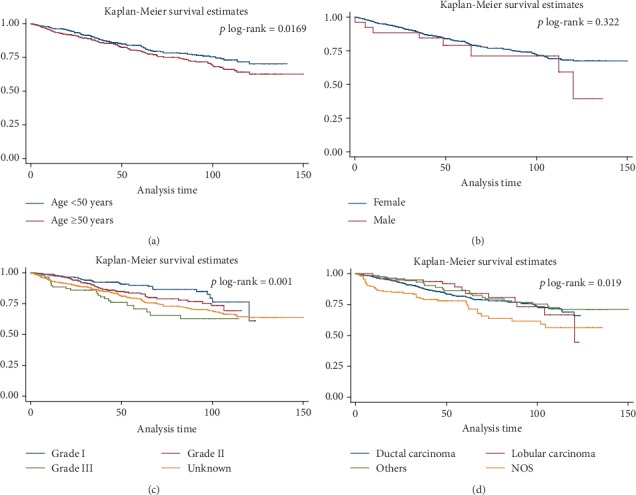
Kaplan-Meier survival curve, results for the test of equality of breast cancer-specific survival functions for the different variables in Northwest of Iran, between 2007 and 2016 in both sexes (a) age groups, (b) sex groups, (c) grades, and (d) morphologic types.

The log-rank test did not show a significant difference for survival status between patients aged <50 and those ≥50 years (*p* = 0.07), and between males and females (*p* = 0.32). There was a significant difference in survival by tumor morphology (*p* = 0.02) and tumor grade (*p* < 0.001) (Figure 1).

Univariate Cox regression analysis showed that male patients had about 1.4 times higher death hazard compared to women (HR = 1.4; 95% CI: 0.7-2.9). Older breast cancer patients (≥50 years) had 1.3 times higher risk when compared to under 50 subjects (HR = 1.3; 95% CI: 0.9-1.6). Patients with higher-grade tumors had higher mortality compared to the grade I tumors (HR = 1.5; 95% CI: 0.9-2.3 for grade II, and HR = 2.5; 95% CI: 1.5-4.4 for grade III tumors). According to the multivariate Cox regression analysis, older patient (≥50 years) had significantly worse survival (HR = 1.3; 95% CI: 1.0-1.6), and higher grades had still significant worse prognosis than grade I tumors (HR = 1.6; 95% CI: 01.0-2.4 for grade II, and HR = 2.6; 95% CI: 1.59-4.4 for grade III tumors) (Table 2).

### 3.2. Trends in Incidence

Trend analysis was performed on the 4989 registered breast cancer cases over the study period (2007-2016). The ASIRs increased from 11.3 to 18.9 (per 100,000) with an APC of 5.4 percent in both sexes. In women, the ASIRs increased from 21.7 to 36.9 (per 100,000) with an overall APC of 5.5 percent and an average AAPC of 6.1 percent. The increase was most obvious during 2014 and 2016 (APC = 16.9 percent). By contrast, in men, we noticed a different pattern of the trend for the incidence of breast cancer indicating that the ASIR decreased from 0.7 to 0.5 with an overall APC of -0.6 percent. The decline in the ASIRs trend among males was mostly noticeable between 2014 and 2016 with an APC of -29.3 percent (Table 3).

## 4. Discussion

In this study, we provided the breast cancer-specific survival proportions and mortality rates, and trends in a northwestern region of Iran based on the data from our regional cancer surveillance system. A relatively better breast cancer-specific survival proportions and mortality rates in the area were observed compared to the overall breast cancer-specific survival proportions in the country. However, it is still poor compared to the developed countries.

Breast cancer is the most frequent cancer in women, and the second most common cancer across the globe [1]. Reports showed that breast cancer was the third most common cancer in the study region of Iran [14]. The highest occurrence has been reported from the United States and Western Europe, while the lowest rates belong to the East Asia [1, 2]. Iran is among those countries having the increasing trends in both incidence and mortality indices. Those rates are however still low compared to some developing countries [5, 15]. Previous reports from Iran have indicated that there are increasing trends both for incidence and mortality of breast cancer in the country. Population aging, adaptation to western lifestyle, no full-term pregnancy, late age at first pregnancy, lack of breast feeding, hormonal pregnancy control, and obesity might have played a role on these trends. However, recent remarkable improvements in cancer registries and data management should not be ignored in the evaluation of the increasing trends of breast cancer in the country [5, 6, 16–18].

Although early diagnosis and better survival have decreased age-standardized disability-adjusted life years for breast cancer in most high-middle income countries over the last decade [2], patients in many low-income countries (including some African and Asian countries) experiencing small life spans [19, 20].

The CONCORD study reported that the 5-year net survival for breast cancer has steadily increased to almost 80 percent in many countries [21]. There are however still remarkable differences between various countries. For instance, breast cancer-specific survival proportions of 81–86 percent have been reported from England, Belgium, Canada, the United States, and Italy whereas the similar figures are much lower in countries including Malaysia (68 percent), India (60 percent), Mongolia (57 percent), and South Africa (53 percent) [21]. These differences may be occurring because of the limited oncology services and treatments, and lack of early detection programs and screening facilities [22]. A recent report from Iran showed that the one-, three-, and five-year breast cancer-specific survival proportions were 95.6, 80.8, and 69.5 percent, respectively [23]. However, the survival from breast cancer is generally low in Iran compared to the developed countries indicating that inappropriate treatment modalities might have played a role on this [8, 10, 24–28]. Some studies, too, showed that tumor size, lymph node involvement, tumor grade, socioeconomic status, and inheritance factors are of the main factors associated with breast cancer-specific survival [16, 29–31].

Cancer registries provide essential information for prevention purposes at community levels. The EA-PBCR is a newly established surveillance system in the northwest region of Iran. Although EA-PBCR is now registering all cancer cases in the population at the time of diagnosis, almost two-third of follow-up data are not accessible for survival purposes. This was a weak point in this study needing an attention for improvement for future studies.

## 5. Conclusion

Compared to previous reports, we observed relatively better breast cancer-specific survival proportions and mortality rates in the region. Population-based campaigns and awareness programs for early detection of breast cancer in the area will further improve the survival status for those diagnosed with breast cancer in the population.

## Figures and Tables

**Table 1:** Main characteristics of breast cancer patients (total and cases with follow-up) in Northwest of Iran, between 2007 and 2016.

Characteristic		Total cases (*n* = 4,989)	Cases with follow-up (*n* = 1335)
Age	Mean (SD)	50.4 (SD = 12.9)	50.6 (SD = 12.6)
	Range	21-98	23-93
	<50 years of age	2299 (46.1%)	658 (49.3%)
	≥50 years of age	2689 (53.9%)	677 (50.7%)

Sex	Male	104 (2.1%)	26 (1.9%)
	Female	4885 (97.9%)	1309 (98.1%)

Morphology	Ductal carcinoma	3096 (62.1%)	1036 (77.6%)
	Lobular carcinoma	585 (11.7%)	81 (6.1%)
	Others	684 (13.7%)	73 (5.5%)
	NOS	624 (12.5%)	145 (10.9%)

Grade	I	736 (14.6%)	232 (17.4%)
	II	1348 (27.0%)	511 (38.3%)
	III	248 (4.9%)	86 (6.4%)
	Unknown	2657 (53.3%)	506 (37.9%)

**Table 2:** Cox regression analysis of follow-up data of breast cancer patients in Northwest of Iran, between 2007 and 2016.

Variable		Univariate Cox regression					Multivariate Cox regression∗			
		Freq. (percent)	HR†	95% CI		*p* value	HR^∗^	95% CI		*p* value
Age	<50	658 (49.3)	Ref.	—	—	—	Ref.	—	—	—
	≥50	677 (50.7)	1.26	0.98	1.61	0.070	1.28	1.00	1.64	0.050

Sex	Female	1309 (98.1)	Ref.	—	—	—	Ref.	—	—	—
	Male	26 (1.9)	1.42	0.70	2.88	0.325	1.34	0.66	2.73	0.414

Grade	I	232 (17.4)	Ref.	—	—	—	Ref.	—	—	
	II	511 (38.3)	1.52	0.98	2.34	0.059	1.55	1.00	2.40	0.048
	III	86 (6.4)	2.53	1.47	4.36	0.001	2.57	1.49	4.44	0.001
	Unknown	506 (37.9)	1.93	1.28	2.91	0.002	1.98	1.27	3.07	0.002
						0.001∗∗				

Morphology	DC	1036 (77.6)	Ref.	—	—	—	Ref.	—	—	—
	LC	81 (6.1)	0.86	0.50	1.49	0.598	0.73	0.42	1.306	0.288
	Others	73 (5.5)	0.86	0.54	1.37	0.522	0.70	0.43	1.15	0.163
	NOS	145 (10.9)	1.63	1.16	2.29	0.005	1.36	0.92	1.99	0.119
						0.032∗∗				0.001∗∗

†Unadjusted Hazard Ratio; ∗Adjusted Hazard Ratio; ∗∗Total *p* Value. HR: hazard ratio; CI: confidence interval.

**Table 3:** Trend analysis of age-standardized incidence rates (ASIRs), breast cancer patients in Northwest of Iran, between 2007 and 2016 in both sexes (per 100,000).

	0 joinpoint			1 joinpoint						0 joinpoint		
	2007-2016			2007-2014			2014-2016			2007-2016		
	APC^∗^	CI†	*p* value	APC	CI	*p* value	APC	CI	*p* value	AAPC††	CI	*p* value
Both	5.4	1.1−9.9	0.823	3.3	-6.3−13.9	0.823	15.8	-36−109.5	0.823	5.4	1.1−9.9	0.823
Female	5.5	1.1−10.1	0.777	3.2	-6.6−13.9	0.777	16.9	-35.9−113	0.777	6.1	-5.7−19.3	0.300
Male	-0.6	-7.3−9.2	0.268	6.1	-4.9−18.5	0.268	-29.3	-71.1−72.9	0.268	0.6	-7.3−9.2	0.268

^∗^APC: annual percentage change. †CI: confidence interval. ††AAPC: Average Annual Percentage Change.

## Data Availability

The datasets analyzed and presented in this study are available from the corresponding authors on a reasonable request.
