# Corrigendum to “Breast Cancer Survival and Incidence: 10 Years Cancer Registry Data in the Northwest, Iran”

**DOI:** 10.1155/2021/2604819

**Published:** 2021-02-20

**Authors:** Roya Dolatkhah, Mohammad Hossein Somi, Mohammad Asghari Jafarabadi, Mehrnaz Hosseinalifam, Sepideh Sepahi, Mina Belalzadeh, Marzieh Nezamdoust, Saeed Dastgiri

**Affiliations:** ^1^Hematology and Oncology Research Center, Tabriz University of Medical Sciences, Tabriz, Iran; ^2^Liver and Gastrointestinal Diseases Research Center, Tabriz University of Medical Sciences, Tabriz, Iran; ^3^Road Traffic Injury Research Center, Tabriz University of Medical Sciences, Tabriz, Iran; ^4^Tabriz University of Medical Sciences, Tabriz, Iran; ^5^Cancer Registry Office, Liver and Gastrointestinal Diseases Research Center, Tabriz University of Medical Sciences, Tabriz, Iran; ^6^Tabriz Health Services Management Research Center, Tabriz University of Medical Sciences, Tabriz, Iran

In the article titled “Breast Cancer Survival and Incidence: 10 Years Cancer Registry Data in the Northwest, Iran” [1], there was an error in “Figure 1(a).” The *p* value has been corrected from *p* log − rank = 0.0169 to *p* log − rank = 0.0692. The corrected figure is shown below.

## Figures and Tables

**Figure 1:**
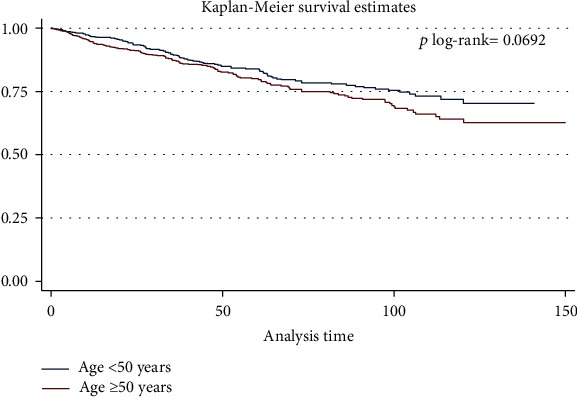
Kaplan-Meier survival curve, results for the test of equality of breast cancer-specific survival functions for the different variables in Northwest of Iran, between 2007 and 2016 in both sexes (a) age groups.

## References

[1] Dolatkhah R., Somi M. H., Jafarabadi M. A. (2020). Breast Cancer Survival and Incidence: 10 Years Cancer Registry Data in the Northwest, Iran. *International Journal of Breast Cancer*.

